# Bench to bedside: it’s all about the model

**DOI:** 10.1186/scrt400

**Published:** 2014-01-20

**Authors:** Mandi J Lopez

**Affiliations:** 1 Laboratory for Equine and Comparative Orthopedic Research, Veterinary Clinical Sciences Department, School of Veterinary Medicine, Louisiana State University, Skip Bertman Drive, Baton Rouge, LA 70803, USA

## Abstract

Continual improvement and targeting of large animal models with contemporary technology augment their accuracy and value. New discoveries within the models contribute to reliable results with reduced variability. Despite the importance of large animal stem cell models to biomedical advances, the knowledge base surrounding them is relatively limited compared with that of human and rodents. The series of investigations presented by Niada and colleagues helps to meet this essential element of large animal models with information about *in vitro* behavior of porcine adipose-derived stem cells from two different harvest sites as well as their responses to implant materials and porcine serum.

See related research by Niada *et al*., http://stemcellres.com/content/4/6/148

## 

Large animal models of human musculoskeletal and dental disorders are invaluable to preclinical safety and efficacy testing prior to advancing novel therapies to clinical trials. The article by Niada and colleagues [1] in a recent issue of *Stem Cell Research & Therapy* provides information about *in vitro* behavior of adipose-derived stem cells (ASCs) from two different harvest sites in a porcine model. Effects of implant materials, titanium and silicon carbide coating, on ASC osteogenesis and porcine serum on cell proliferation are also assessed. Animal model validation that includes thorough characterization of the proposed therapy and confirms accuracy for the target clinical condition is critical to outcome information that benefits human and veterinary patients [2]. The results of the studies described in the article impact future studies in the area by contributing to the current understanding of porcine ASCs from a facial harvest site.

The pig is an established animal model for dental, oral, and maxillofacial research and preclinical testing. In fact, it is a model of choice for novel implant design testing for a number of reasons [3]. Porcine bone anatomy, morphology, healing, and remodeling are similar to those of humans [4]. The rate of bone regeneration in pigs (1.2-1.5 mm/day) is reportedly closer to that in humans (1.0-1.5 mm/day) than dogs (1.5-2.0 mm/day) [3]. Deciduous and permanent tooth morphology and periodontal tissue anatomy of miniature pigs are similar to those of humans [5]. Porcine models are a major focus of regenerative medicine studies using stem cells harvested from numerous harvest sites, including adipose tissue [6]. Although there are recognized limitations of animal models for human conditions from unavoidable differences among species, there is no question that animal models are central to veterinary and human medical advances. Focused research efforts to improve upon the models will increase the value and potential impact of data derived from studies that use them.

Characterization of ASCs from distinct adipose tissue harvest sites is vital to clinical translation of the technology. Adipose tissue harvested during elective liposuction is a common source of ASCs for autologous administration [7]. However, the procedure is not necessarily practical for all patients. Additionally, adipose tissue deposition varies in ASC quality and quantity among depots in the same individual and for the same depot between individuals, human and animal [1,8]. Current knowledge supports the view that ASCs isolated from orthotopic locations may have advantages over those isolated from tissue distant to the treatment site [9]. Differences in ASC behavior among species also limit extrapolation of information among them [8]. It is therefore critical to carefully characterize and identify species- and depot-specific ASCs to appropriately qualify results and conclusions. This is also important for generation of species-compatible implants to avoid xenograft complications. Studies such as that by Niada and colleagues address several of these important issues.

A recent position statement by the International Federation for Adipose Therapeutics and Science and the International Society for Cellular Therapy provides minimal criteria to define stromal vascular fraction cells and ASCs [7]. Basic characterization of cultured ASCs includes phenotyping with flow cytometry and confirmation of the ability of the cells to differentiate into osteoblastic, chondrocytic, and adipocytic lineages. Current standards support confirmation of cell differentiation using target gene and protein expression quantitation versus histochemical analysis. The study by Niada and colleagues incorporates many of the recommendations. Unfortunately, passage 4 cells tested with monoclonal antibodies against CD14, CD45, CD73, CD90, CD105, and CD271 showed that cells were CD90^+^, CD271^-^, CD45^-^, and CD14^-^. There was no cross-reactivity of the CD73 and CD105 antibodies. Shortages of monoclonal antibodies directed against animal cell surface markers can limit the ability to determine stem cell phenotype in large animal models [10]. When it is necessary to use non-species-specific antibodies, confirmation of reactivity with the target species protein is necessary for valid results. Standardized, rigorous, and consistent phenotyping of ASCs in animal models is critical to robust results that support repeatability and the greatest potential for application across species.

*In vitro* knowledge of interactions between stem cells and implant materials is required to anticipate the *in vivo* response of exogenous and native stem cells. *In vitro* culture conditions that closely mimic the natural environment of the clinical target site provide valuable insight for preclinical models. Porcine ASCs cultured on titanium, silicon carbide coating, and plastic were evaluated for total protein concentration and calcium-rich deposits after 21 days of culture in osteogenic or basal medium in the study. Based on staining, there was greater deposition by ASCs on titanium discs compared with plastic when cultured in both basal and osteogenic media. Total protein levels were less supportive of differences among culture surfaces. A more in-depth evaluation of porcine ASC osteogenic differentiation on implant materials that includes protein and gene expression at several time points will provide opportunities for detailed comparisons among parallel studies with ASCs from different species as well as among investigations using porcine models [11].

Alternatives to fetal bovine serum (FBS) supplementation of basal medium for *ex vivo* expansion of stem cells are an area of significant interest. Despite efforts to characterize FBS, there is variability among preparations, and there is always the potential for xenogeneic proteins to affect cell behavior [12]. Quality and safety of stem cells intended for therapeutic application will be enhanced by expansion under xeno-free conditions. Results from porcine ASCs cultured in heterologous or homologous serum do not support use of species-specific serum in the study by Niada and colleagues. However, serum quality compounded by a relatively low concentration in the medium may have impacted the outcomes. Outcomes were limited to cell proliferation and morphology in basal medium containing 5% heterologous or homologous porcine serum or 10% FBS. Other studies suggest that higher levels of adult serum are necessary to support the same *in vitro* cell behavior observed at lower FBS levels [13]. Continued, in-depth efforts toward development of xeno-free culture conditions are crucial to continued progress in this area.

Optimization of preclinical animal models facilitates design and implementation of regenerative therapies, and porcine models are established for a large number of oromaxillary and dental procedures. Harvest of ASCs from the buccal fat pad for orthotopic administration is a logical extension of current surgical procedures. Information about porcine buccal fat pad ASCs is limited despite the model’s importance to continued medical advances in the area. Differences among adipose tissue depots and species require individual characterization of ASCs among them. The results of the study by Niada and colleagues contribute to the current understanding of porcine buccal adipose tissue ASCs to support future investigations in this important animal model.

## Abbreviations

ASC: Adipose-derived stem cell; FBS: Fetal bovine serum.

## Competing interests

The author declares that she has no competing interests.
